# Are we ready to think differently about setting PEEP?

**DOI:** 10.1186/s13054-022-04058-1

**Published:** 2022-07-19

**Authors:** Matthew E. Cove, Michael R. Pinsky, John J. Marini

**Affiliations:** 1 Department of Medicine, National University Singapore, NUHS Tower Block Level 10, 1E Kent Ridge Road, Singapore, 119228 Singapore; 2 Department of Critical Care Medicine, 638 Scaife Hall, University of Pittsburgh, 3550 Terrace Street, Pittsburgh, PA 15261 USA; 3 Pulmonary and Critical Care Medicine, Regions Hospital and University of Minnesota, Minneapolis/Saint Paul, MN USA

In stark contrast to the undisputed mortality benefit of lower tidal volume ventilation in patients with acute respiratory distress syndrome (ARDS) [1], the best strategy to determine optimal positive end expiratory pressure (PEEP) remains an unresolved question [2–6]. It is an important question because conventional understanding predicts the right PEEP will maintain recruitment of mechanically unstable alveoli, improving both oxygenation and lung compliance. Improved compliance, combined with lower ventilation volumes, maximizes lung protection by limiting tidal airway pressure changes.

Oxygenation is a convenient target for determining PEEP, and protocols directing clinicians to increase PEEP in a stepwise fashion, based on the fraction of inspirated oxygen (FiO_2_) required to maintain arterial oxygen levels within a specified range, have been used in several studies and guidelines [1–3]. Increasing PEEP to “chase” FiO_2_ requirements in this manner is simple, reproducible, and in the absence of a superior strategy [2–6], commonly practiced. However, it assumes the dominant mechanism of hypoxemia is alveolar collapse and an associated reduction in compliance, where increasing PEEP increases recruitment of *functional* lung units. These assumptions fail when patients meet ARDS criteria and the dominant mechanism of hypoxemia is not alveolar collapse, because hypoxemia may coexist with minimally impaired lung compliance. While such patients may only represent one end of the compliance spectrum in ARDS [7], increasing PEEP to “chase” FiO_2_ requirements in this setting leads to the use of ever higher PEEP, even though relatively few *functional* lung units are re-opened.

This mechanistic distinction is important. When increasing PEEP does not recruit *functional* lung units and improve pulmonary compliance, it will increase lung distention and energy transfer to the pulmonary-parenchymal matrix [8]. This raises the risk of lung injury, dead-space formation, pneumothorax, and detrimental hemodynamic consequences. Oxygenation measures are not sensitive to this; increasing PEEP elevates mean airway pressure, and Henry’s law predicts this also increases the partial pressure of oxygen (PaO_2_) to FiO_2_ (P:F) ratio regardless of whether *functional* lung units are recruited, at least until cardiac output becomes impaired. This would be of no consequence if the coexistence of hypoxemia and minimally impaired compliance was exceedingly rare in ARDS. However, this is not the case because the ARDS definition only accounts for P:F ratio, a measure of oxygenation, not compliance. Therefore, although mean lung compliance in ARDS cohorts is usually low, the range is wide and some patients may experience only mild compliance reductions [7].

Patients with COVID-19 are an example of this phenomenon. The mechanisms of hypoxemia in the early phases of disease appear to be driven more by pulmonary endothelial dysfunction than by collapse of functional alveoli. This is because the virus gains cellular entry via the angiotensin-converting enzyme II receptor, which is not only present in the lung epithelium, but also abundantly present in vascular endothelium and arterial smooth muscle cells [9]. Therefore, as well as causing pneumonia, the virus incites inflammation of the pulmonary vasculature leading to a ‘*V*A/*Q*’ mismatch and P:F ratio that is out of proportion to the change in pulmonary mechanics [9]. Under these circumstances, “chasing” FiO_2_ with PEEP might lead to continued upward titration of PEEP even though few *functional* lung units are re-opened, potentially causing harm.

That different ARDS patients might respond differently to PEEP was documented well before COVID-19. In 2014 Calfee and colleagues identified two ARDS sub-phenotypes based on inflammatory biomarkers [10]. They demonstrated that higher PEEP reduced mortality in patients with a hyper-inflammatory sub-phenotype and increased mortality in those with a hypo-inflammatory sub-phenotype. Although the effect of PEEP on different ARDS sub-phenotypes of lung compliance has not been described [7], it is harmful in other settings where hypoxemia leads to the use of high PEEP levels despite relatively conserved compliance [11]. Given the inherent pitfalls of an oxygenation-based PEEP strategy, perhaps a more physiologic approach is required.

Setting PEEP to target optimal compliance can overcome these pitfalls. When an increase in PEEP benefits any patient, *functional* lung units are recruited and compliance increases. If an increase in PEEP is unhelpful, few *functional* units will be recruited and compliance will remain unchanged or decrease, even though the P:F ratio may still increase. In the modern ICU, compliance is easily determined. The least-squares fit procedure determines breath-to-breath dynamic respiratory compliance from the monitored airway pressure, volume and flow [12], without an end inspiratory breath hold. In the absence of real-time dynamic compliance, using PEEP to optimize driving pressure may be a suitable surrogate. In spontaneously breathing patients, reliable compliance measurements can be provided with modes like proportional assist ventilation [13].

Using these real-time compliance measures, PEEP can be titrated upward or downward, and the effect on compliance observed [14]. If compliance increases, the new PEEP is more optimal, and if compliance decreases, the new PEEP is either too high or too low. When compliance is unchanged after titrating PEEP upward, a clinical judgment on the likelihood of recruiting *functional* lung units with higher PEEP is required. The clinician’s goal should be achieving the highest possible compliance with the lowest possible PEEP, rather than a specific compliance target, since this will vary from patient-to-patient and is sensitive to other commonly used ventilator settings. Optimizing PEEP in this manner also reduces dead-space ventilation, and while this is a more complex bedside measurement, the ventilatory ratio closely corresponds and can be simply tracked [15], helping confirm whether the new PEEP is more optimal.

Prescribing PEEP based on oxygen requirements is a “one-size-fits-all” approach, destined to help some patients, while exposing others to harm. Alternatively, using modern monitoring tools to optimize PEEP based on measures of pulmonary physiology, such as compliance, allows clinicians to better personalize ventilator settings to help all patients. Although we still lack high-quality clinical trials demonstrating that setting PEEP based on respiratory compliance measures is superior to using measures of oxygenation, we hypothesize that future ARDS management strategies which optimize PEEP based on patient physiology, while observing threshold limits for variables like plateau pressure and driving pressure, will further improve outcomes for all ARDS patients.

## Data Availability

Not applicable.
